# Integrated proteomic analysis of *Brachypodium distachyon* roots and leaves reveals a synergistic network in the response to drought stress and recovery

**DOI:** 10.1038/srep46183

**Published:** 2017-04-07

**Authors:** Yanwei Bian, Xiong Deng, Xing Yan, Jiaxing Zhou, Linlin Yuan, Yueming Yan

**Affiliations:** 1 College of Life Science, Capital Normal University, 100048 Beijing, China

## Abstract

In this study, we performed the first integrated physiological and proteomic analysis of the response to drought and recovery from drought, using *Brachypodium distachyon* L. Roots and leaves. Drought stress resulted in leaves curling, root tips becoming darker in color and significant changes in some physiological parameters. Two-dimensional difference gel electrophoresis (2D-DIGE) identified 78 and 98 differentially accumulated protein (DAP) spots representing 68 and 73 unique proteins responding to drought stress and/or recovery in roots and leaves, respectively. Differences between the root and leaf proteome were most marked for photosynthesis, energy metabolism, and protein metabolism. In particular, some DAPs involved in energy and protein metabolism had contrasting accumulation patterns in roots and leaves. Protein-protein interaction (PPI) analysis of roots and leaves revealed complex protein interaction networks that can generate synergistic responses to drought stress and during recovery from drought. Transcript analysis using quantitative real-time polymerase chain reaction (qRT-PCR) validated the differential expression of key proteins involved in the PPI network. Our integrated physiological and proteomic analysis provides evidence for a synergistic network involved in responses to drought and active during recovery from drought, in *Brachypodium* roots and leaves.

Plants encounter a variety of biotic and abiotic stresses during growth^1^. These stresses unbalance cellular homeostasis and lead to morphological, physiological, and molecular changes^2^. These changes have a negative impact on survival and biomass production, and can reduce final yield by up to 82%^3^. Global warming and climate change may also be exacerbating the effects of abiotic stresses on crop production in many areas of the world today. Previous reports have suggested that a temperature increase of 1 °C can produce a decrease in yield of up to 10%^4^. Drought in particular, severely impairs plant growth and development and limits crop productivity more than any other environmental factor^5^,^6^. As the climate continues to change, drought may become a more frequent and cause severe problem^7^.

Drought stress induces a range of physiological and biochemical responses in plants. Under drought conditions, the plant root cap produces abscisic acid (ABA), which mediates stomatal closure. This in turn, suppresses cell growth, photosynthetic efficiency, and respiration^8^,^9^,^10^. Recently, ABA and stomata have been implicated in the regulation of systemic responses to abiotic stresses^11^. In addition, plants generate toxic ions and reactive oxygen species (ROS) that can decrease enzyme activities and damage essential proteins^12^. The generation of ROS occurs early in the response to water-stress, and ROS are key secondary messengers triggering subsequent defensive measures in plants^1^,^13^,^14^,^15^. Plants have developed a sophisticated and elaborate system for scavenging high levels of ROS using antioxidant enzymes that include superoxide dismutase (SOD), catalase (CAT), peroxidase (POD), and ascorbate peroxidase (APX). They also use antioxidant compounds, including ascorbate and glutathione^16^,^17^,^18^. Drought stress produces changes in the expression of a related set of genes, inhibiting the synthesis of normal proteins and resulting in the production of stress-specific proteins^19^. Recent reports suggest ROS can enhance ABA biosynthesis or inhibit its degradation, thereby increasing free ABA levels^20^,^21^,^22^,^23^. Increased ROS production might enhance ABA accumulation, while higher levels of ABA could generate more ROS production in guard cells, creating a positive feedback loop and mediating stomatal closure^24^. In addition, drought also leads to changes in plant calcium (Ca^2+^) concentrations that can trigger signaling cascades^25^, and high concentrations of ROS can produce synergistic responses in both plant roots and leaves^26^.

To date, the molecular basis of drought responses and tolerance in different plant species has been investigated by numerous proteomic studies and supplemented by a rapidly advancing understanding of genomics in many species that include Arabidopsis^27^, rice^28^, wheat^29^,^30^, soybean^31^, and napus^32^. However, most of the proteomic analyses have focused on individual organs and their results cannot reflect any synergistic responses involving different organs acting together to deal with adverse environmental conditions. Therefore, it is important to perform an integrated proteomic analysis using different plant organs to gain a better understanding of how plants adapt to drought stress.

*Brachypodium distachyon* L. is a model plant related to economically important crop species that include wheat and barley, and also to several potential biofuel grasses that include switchgrass^33^. The sequencing and annotation of the complete *B. distachyon* Bd21 genome^34^ can now facilitate proteomic studies investigating biotic and abiotic stress mechanisms. In this study, we performed the first integrated physiological and proteomic analysis of *B. distachyon* seedling roots and leaves responding to drought stress and also during the subsequent drought recovery period. Our results reveal a complex regulatory network triggered by drought, and they provide new insight into the molecular mechanisms of plant drought responses.

## Results

### Morphological and physiological changes in Bd21 seedlings in response to drought stress and during subsequent recovery

**Figure 1:**
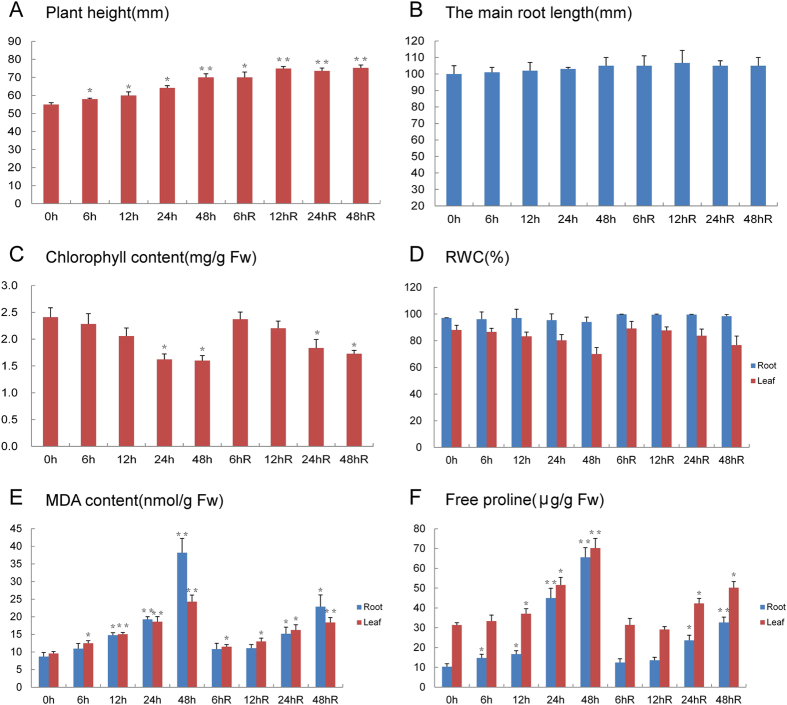
Physiological parameters changes in Bd21 roots and leaves under drought stress and recovery. **(A)** The plant height analysis of Bd21 under drought stress and recovery; **(B)** The main root length analysis of Bd21 root under drought stress and recovery; **(C)** Chlorophyll content analysis of Bd21 leaf and root under drought stress and recovery; **(D)** Relativewater content (RWC) analysis of Bd21root and leaf under drought stress and recovery; **(E)** Malonaldehyde (MDA) content analysis of Bd21root and leafunder drought stress and recovery; **(F)** Free proline content analysis of Bd21 root and leafunder drought stress and recovery. Error bars indicate standard errors of three biological replicates. Statistically significant differences compared to the control were calculated based on an independent Student’s t-tests: ^*^P < 0.05; ^**^P < 0.01.

Significant morphological and physiological changes took place in seedlings grown under drought conditions and during the subsequent recovery process. Figure S1 demonstrates that during a prolonged period of drought stress the plant root tips gradually became darker in color. Leaves began to curl after 24 h and leaf curling became very obvious after 48 h. During recovery from drought, seedlings were able to regain their normal morphology, but plant height increased only slowly and root length did not change significantly (Fig. 1).

Physiological analysis demonstrated that leaf chlorophyll content decreased significantly in response to drought stress and after a treatment of 24 h was unable to return to its pre-drought treatment level. Changes in leaf relative water content (RWC) were similar to those of chlorophyll content, but root RWC was not altered significantly during drought stress or the subsequent recovery period. The malondialdehyde (MDA) content of both roots and leaves increased significantly in response to drought stress. In seedlings from the 6 and 12 hours of recovery (hR) seedling groups, MDA levels returned to normal, while in seedlings from the 24 and 48 hR groups they did not. The trend for changes in proline content was similar to that of MDA in both roots and leaves. If the drought stress period exceeded 24 h, physiological changes in the plants were difficult to reverse.

### Proteome expression profiles and multivariate analysis of drought stress responses and drought stress recovery

**Figure 2:**
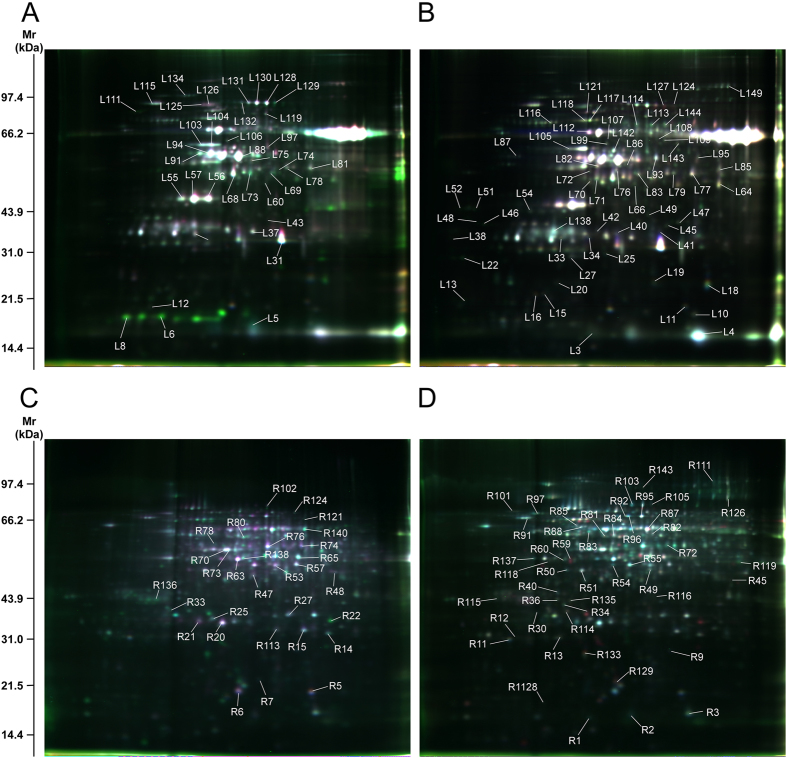
2D-DIGE images of Bd21 roots and leaves under drought stress and recovery. **(A)** Leaf gel 1; **(B)** Leaf gel 4; **(C)** Root gel 1; **(D)** Root gel 4. Numbered lines indicate spots that were identified by MALDI-TOF/TOF-MS and significantly regulated under drought stress and recovery.

Two-dimensional difference gel electrophoresis (2D-DIGE) demonstrated that changes in 143 and 149 protein species had occurred at 24 h and 24 hR in roots and leaves, respectively (Fig. 2). We detected the dynamic expression profiles of these differentially accumulated proteins at 0, 6, 12, 24, and 48 h, and 6, 12, 24, and 48 hR (DAPs) using two-dimensional electrophoresis (2-DE). All of the DAP spots were reproducibly detected and matched using the 2-DE profiles from roots and leaves, respectively. Representative 2D-DIGE and 2-DE gels are shown in Fig. 2 and in Supplementary Figs S2 and S3.

**Figure 3:**
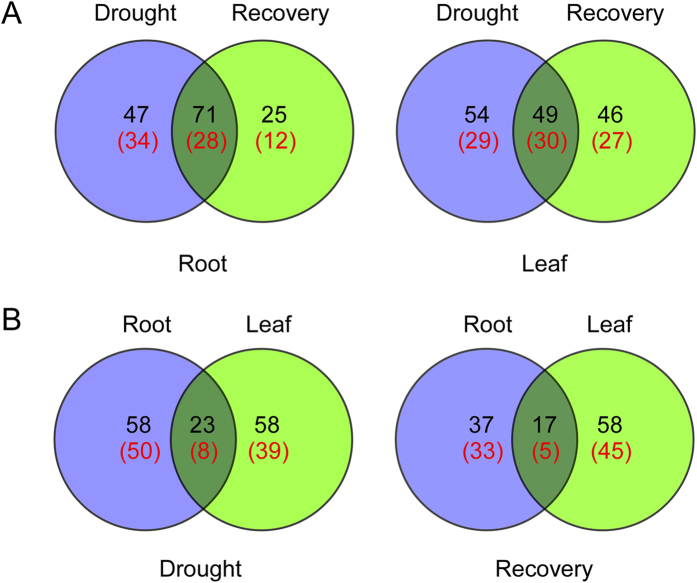
Venn diagram analysis of DAP spots in Bd21 roots and leaves under drought stress and recovery. **(A)** Venn diagram analysis of DAP spots in roots and leaves; **(B)** Venn diagram analysis of DAP spots under under drought stress and recovery. The red number represents the number of unique protein species identified.

The number of DAP spots and their overlapping relationships following drought treatment and recovery are shown as Venn diagrams in Fig. 3A. In roots, 47 DAP spots exhibited significantly changed abundance in response to drought stress. This was many more than the 25 DAP spots that were differentially accumulated during recovery in roots, and similar to the number of DAP spots (95) that accumulated differentially during recovery in leaves. These results suggest that the drought response of plants continues during the subsequent recovery periods. Under drought stress, the numbers of DAP spots in roots and leaves were similar, but when the drought treatment was removed there were far more DAP spots in leaves (75) than in roots (54), we speculate that during recovery, metabolic activity in leaves was greater than in roots. An analysis using Venn diagrams of unique protein species identified under drought stress and during the subsequent recovery period provided similar results (Fig. 3B).

To visualize co-accumulation among DAP spots, we performed a hierarchical cluster analysis to analyze the proteome dataset and reveal changes in protein expression in each tissue during drought treatment and the subsequent recovery period. Two hierarchical clusters corresponding to root (Fig. S4A) and leaf (Fig. S4B) samples were constructed. The DAP spots from the root and leaf samples were each divided into six clusters. In roots, cluster 1 included DAP spots with increased accumulation after 6 and 24 h drought treatment, but decreased accumulation during the subsequent recovery period. The DAP spots in clusters 2 and 3 showed increased accumulation in the drought treatment group, but decreased accumulation in the recovery group. In particular, the DAP spots in cluster 3 showed increased accumulation at both 6 and 24 hR. Cluster 4 included DAP spots with increased accumulation in the drought treatment group but decreased accumulation in the recovery group, and also greater accumulation at 12 h, 24 h, 6 hR, and 24 hR. The DAP spots in clusters 5 and 6 had diverse accumulation patterns in both treatment and recovery groups (Fig. S4A).

In leaves, the cluster 1 DAP spots showed increased accumulation in the drought treatment group, but decreased accumulation in the recovery group. Those from cluster 2 had increased accumulation in both treatment and recovery groups with greater accumulation at 6 and 48 hR, while cluster 3 DAP spots had decreased accumulation in the drought treatment group and increased accumulation in the recovery group. Cluster 4, like cluster 2, included DAP spots with increased accumulation in both treatment and recovery groups and these had greater accumulation at 24 and 48 h. The cluster 5 DAP spots had increased to decreased accumulation in the drought treatment group and decreased to increased accumulation in the recovery group. Cluster 6 DAP spots had increased accumulation in the drought treatment group but decreased accumulation at 48 h, and no obvious accumulation pattern in the recovery group (Fig. S4B).

**Figure 4:**
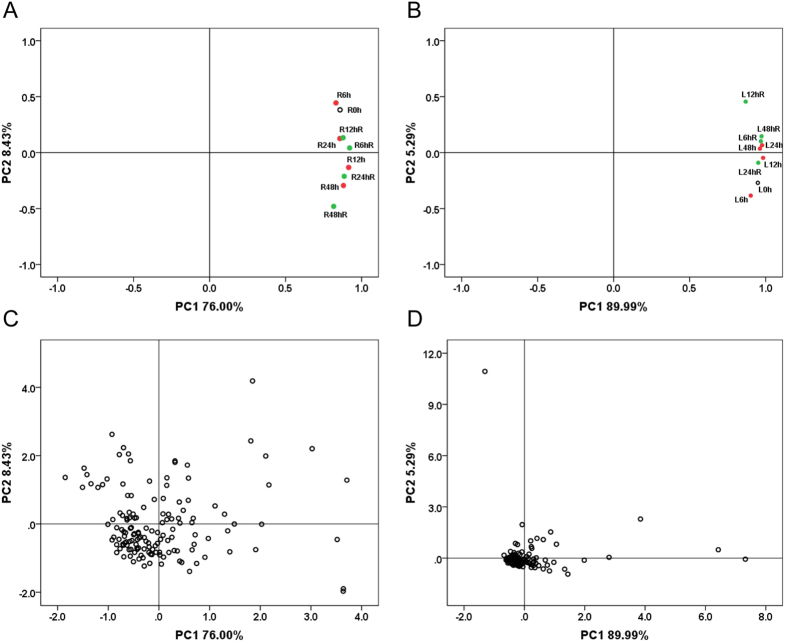
PCA of protein samples and DAP spots. **(A)** PCA of individual protein samples in roots; **(B)** PCA of individual protein samples in leaves; **(C)** PCA of 143 DAP spots in roots; **(D)** PCA of 149 DAP spots in leaves. L0h, L6h, L12h, L24h, L48h, L6hR, L12hR, L24hR and L48hR represent the leaf proteinsamples under drought stress and recovery, respectively; R0h, R6h, R12h, R24h, R48h, R6hR, R12hR, R24hR and R48hR represent the root proteinsamples under drought stress and recovery, respectively.

Principal component analysis (PCA) can be used to identify affected protein species, potential outliers, and clusters^35^,^36^. Figure 4 demonstrates that protein samples from leaves of the drought treatment seedling group (L6 h, L12 h, L24 h, and L48 h) and the recovery seedling group (L6 hR, L12 hR, L24 hR, and L48 hR) differ from the controls (0 h) in PCA plots (Fig. 4A). This indicates that the leaf proteome was significantly altered under both drought and subsequent recovery conditions compared with a control sample. Similarly, protein samples from roots of the drought treatment (R6 h, R12 h, R24 h, and R48 h) and recovery groups (R6 hR, R12 hR, R24 hR, and R48 hR) also differed from control samples (Fig. 4B). PCA of DAP spots in roots and leaves (Fig. 4C,D) demonstrated that some protein spots were isolated from the others, and that there were more potential outliers in roots than in leaves, suggesting a stronger response to drought stress and during subsequent recovery from drought in roots than in leaves. The isolated protein spots were considered potential outliers and prioritized for MALDI-TOF/TOF-MS analysis. A total of 176 DAP spots were identified using tandem mass spectrometry. Of these, 78 and 98 representing 68 and 74 unique protein species were identified from roots and leaves, respectively (Tables S2 and 3). In addition, 15 DAP spots showed differential accumulation in both roots and leaves (Table S5).

### Functional classification and subcellular localization of DAPs

**Figure 5:**
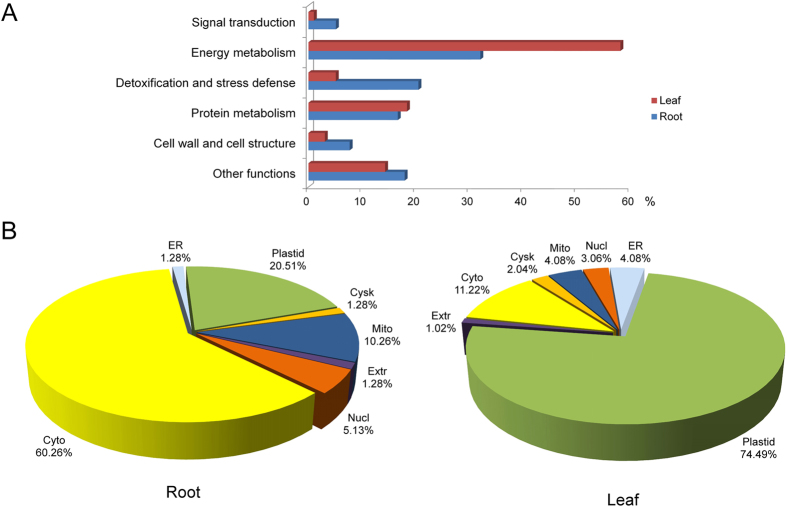
Functional classification and subcellular localization of DAPs from leaves and roots. **(A)** Functional classification; **(B)** Subcellular localization. Cyto, Cytoplasm; Cysk, Cytoskeleton; ER, Endoplasmic Reticulum; Extr, Extracellular; Mito, Mitochondrial; Nucl, Nuclear

All 176 DAPs identified in roots and leaves were classified by function into six categories (Fig. 5A): signal transduction, energy metabolism, detoxification and stress responses, protein metabolism, cell wall and structure, and other functions. The energy metabolism category was the largest and included 25 (32.05%) and 57 (58.16%) DAPs from roots and leaves, respectively. Detoxification and stress response proteins accounted for 20.51% (roots) and 5.1% (leaves), while DAPs involved in protein metabolism accounted for 16.67 and 18.37% from roots and leaves, respectively. Proteins associated with the cell wall/structure comprised 7.69% of those in roots and 3.06% of those in leaves. Only four and one DAP identified from roots and leaves, respectively were related to signal transduction.

The subcellular localization of DAPs identified in roots and leaves was predicted using WoLF PSORT, Plant-mPLoc, and UniprotKB. In leaves, 74.49% of the DAPs were localized in the plastid, while 60.26% of the DAPs from roots were localized in the cytoplasm. In root and leaf samples, 10.26 and 4.08% of the DAPs respectively were localized in mitochondria. In addition, one root DAP (1.28%) was localized in the cytoskeleton and four leaf DAPs (4.08%) were localized to the endoplasmic reticulum (Fig. 5B).

### Protein-protein interaction (PPI) analysis

**Figure 6:**
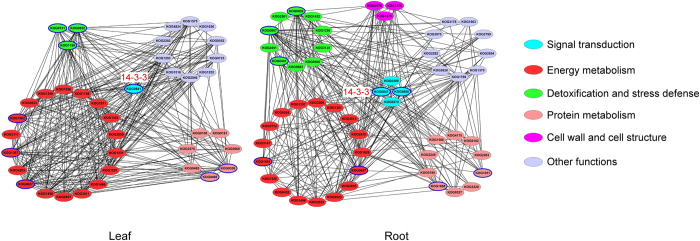
PPI networks under drought stress and recovery in roots and leaves of Bd21. Nodes with bright blue edges represent the important DAPs. KOG0841,14-3-3-like protein A-like; KOG1662,ATP synthase delta chain; KOG1367,Phosphoglycerate kinase; KOG0657,glyceraldehyde-3-phosphate dehydrogenase B; KOG0852,2-Cys peroxiredoxin BAS1; KOG1198,Quinone oxidoreductase-like protein At1g23740; KOG0731,ATP-dependent zinc metalloprotease FTSH 2; KOG0460,elongation factor Tu; KOG0356,Chaperonin CPN60-2; KOG0888,NDPK2; KOG1643,triosephosphate isomerase; KOG0867,glutathione S-transferase 3-like; KOG0441,superoxide dismutase [Cu-Zn] 4A-like; KOG1051,chaperone protein ClpC1; KOG1668,elongation factor 1-beta-like isoform

In plants, proteins do not function in isolation within cells, but as part of a network^37^. In this study, a PPI network was generated to highlight interactions and relationships between different proteins. Figure 6 demonstrates that a large proportion of the proteins identified in both roots and leaves were involved in energy metabolism, while cell wall and cell structure associated proteins were important in the PPI network in roots but not in leaves. We found that signal transduction-related proteins interacted with proteins from other functional groups in both the root and leaf PPI networks, but that these were more prevalent in roots than in leaves. We speculate that signaling processes are more active in roots than in leaves responding to drought. In particular, KOG0841 (14-3-3-like protein A) was involved in interactions with proteins from other functional groups in both roots and leaves, suggesting it has an important signaling role in both organs. Similar results were obtained from Bd21 seedlings subjected to H_2_O_2_ stress^26^.

### Transcript analysis using quantitative real-time polymerase chain reaction (qRT-PCR)

**Figure 7:**
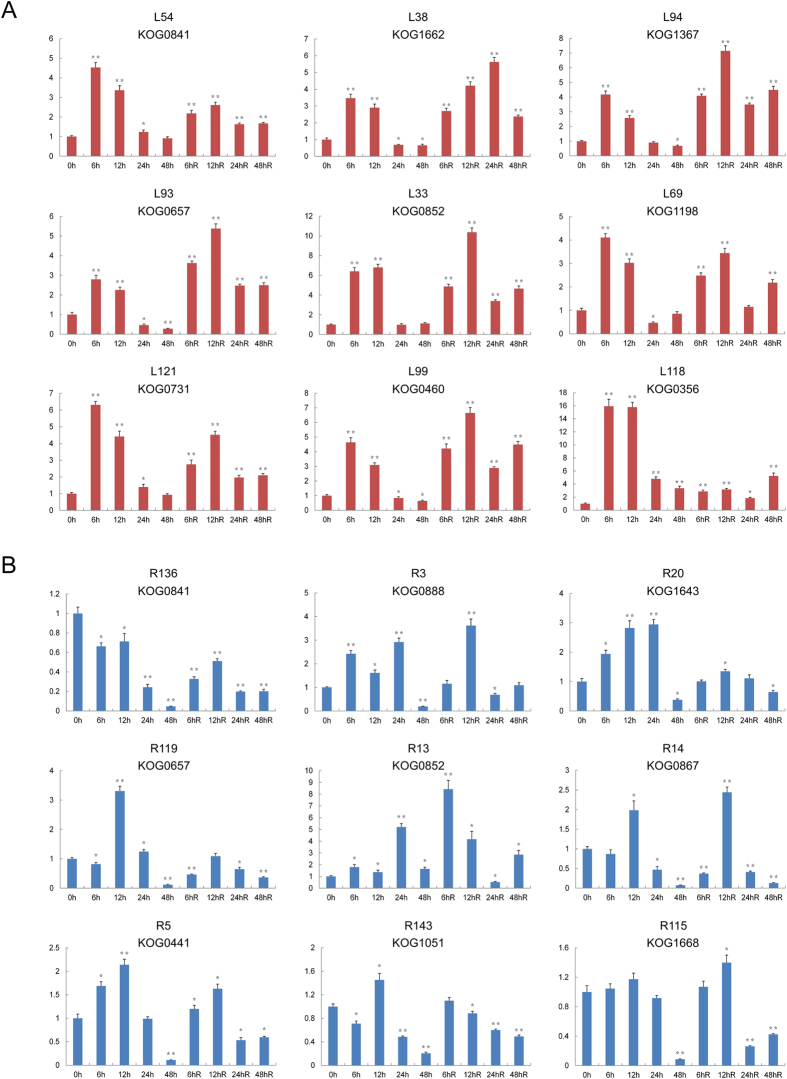
qRT-PCR analysis of representative DAPs in roots and leaves. Statistically significant differences compared to the control were calculated based on an independent Student’s t-tests: *P < 0.05; ^**^P < 0.01.

Based on the proteomic and PPI network data, we selected 18 representative proteins (9 each from roots and leaves) for transcript analysis using qRT-PCR. Of the proteins selected, the 14-3-3-A (KOG0841, L54, and R136) and the 2-Cys peroxiredoxin BAS1 (KOG0852, L33, and R13) proteins had been identified in both the root and leaf analyses, while the other proteins had been identified from either leaves or roots (Fig. 7).

Comparing gene expression at the transcript level with protein synthesis at the translational level demonstrated that seven of the selected proteins (L94, L69, L99, R13, R14, R5, and R115) had strong correlations between their transcript and protein quantities, while three proteins (L121, R36, and R20) showed a similar trend between their transcripts and proteins, and eight proteins (L54, L38, L93, L33, L118, R3, R119, and R143) showed little correlation between the two (Fig. 7 and Table 1).

## Discussion

In this research, we investigated the physiological and proteomic responses to drought stress and subsequent recovery present in *Brachypodium* roots and leaves. A significant number of DAPs were identified in both organs. Here, we discuss the key DAPs and their different functions, together with the synergistic networks that mediate responses active during drought stress and recovery from drought.

### Signal transduction

Plants generate ROS in response to drought, and these ROS function as important regulators of many biological processes including stress responses, hormone signaling, cell growth, and development^13^,^14^,^15^,^38^. In this study, we identified four signal transduction-related proteins in roots and one in leaves (Tables S2 and 3), which increased or decreased significantly in response to drought stress and recovery. We speculate that signaling processes are more active in roots than in leaves under these conditions. In particular, 14-3-3-like protein A (gi|357118054, L54, and R136) was identified from both organs. The 14-3-3 proteins function in protein phosphorylation cascades by associating with specific phospho-sites^39^. They can alter enzyme activity, prevent or induce protein degradation, or affect the subcellular localization of proteins by binding to their targets^40^,^41^,^42^. In addition, 14-3-3 proteins can also activate Ca^2+^-dependent protein kinase (CDPK)^43^, which in turn activates a variety of transcription factors that regulate gene expression^44^. Our results demonstrated that 14-3-3-like protein A accumulated in both roots and leaves in response to drought stress, and during the subsequent recovery period could return to its pre-stress translational level (Table 1). However, it was downregulated at the transcriptional level in roots (Fig. 7). Our recent work also demonstrated that 14-3-3-like protein A transcripts and protein accumulated in response to salt stress in *B. distachyon* leaves^33^, while other 14-3-3 family members including 14-3-3-like proteins GF14-B and GF14-D also showed differential accumulation under H_2_O_2_ stress in both roots and leaves^26^. The PPI network generated in this study demonstrates that 14-3-3 proteins interact with many other proteins that have diverse functions, suggesting 14-3-3 proteins have important roles in defense against drought stress.

Guanine nucleotide-binding protein subunit beta-like protein A, which is also the receptor for activated C kinase 1 A (RACK1A), is a WD-40 type scaffold protein that is highly conserved among eukaryotes. RACK1A is a major component of the RACK1 regulatory proteins that are involved in many signal transduction pathways and regulate developmental and abiotic stress defense-related processes^45^,^46^. In addition, it acts as a novel scaffold protein, binding to the Gβ subunit and functioning in all three tiers of the MAP kinase cascade^30^, interacting with many stress response proteins. It has a major role in promoting crosstalk among these signaling pathways^47^. Our results demonstrated that RACK1A (gi|357132654, R45) accumulation increased in roots responding to drought stress, peaking at 12 h, but that the protein could also return to normal levels (Table 1), suggesting it plays an important role in the early stages of drought resistance.

### Photosynthesis and energy metabolism

The photosynthesis of plants is obviously affected under drought stress and recovery, leaf chlorophyll content decreased significantly in response to drought stress. Our study identified several proteins associated with photosynthesis (Table 1), including chlorophyll a-b binding protein 2 (gi|357159913, L138) and chlorophyll a-b binding protein 8 (gi|357139429, L35), which are involved in the light reaction. These accumulated continuously (Table 1), indicating that the photosynthetic light reaction remained active during drought stress. Three DAPs (L15, L144, and L113) that form part of the ribulose-1, 5-bisphosphate carboxylase/oxygenase large subunit (gi|194033157) were downregulated (Table 1), suggesting that the dark reaction of photosynthesis is inhibited by drought stress.

Plants need to express proteins involved in carbohydrate metabolism to maintain normal growth and development under stress conditions^48^. In this study, many DAPs related to energy metabolism were downregulated in leaves but upregulated in roots (Tables S2 and 3), suggesting that energy metabolism is regulated differently in roots and leaves when plants are responding to drought. In roots, 2, 3-bisphosphoglycerate-independent phosphoglycerate mutase (gi|357125604, R92, R95, and R96), malate dehydrogenase (gi|357146638, R57), and the pyruvate dehydrogenase E1 beta subunit (gi|357148637, R60) which is required to supply the TCA cycle, were all upregulated in response to drought stress but were able to return to normal levels subsequently (Table 1). We speculate that plants must increase their metabolism to provide sufficient ATP for various physiological activities occurring in roots while responding to drought stress.

Several DAPs associated with glycolysis were also identified by our study (Tables S2 and 3). In leaves, DAP L91 was identified as phosphoglycerate kinase (gi|357133147), and was significantly upregulated at 6 h, but downregulated 48 h after the onset of drought stress. Glyceraldehyde-3-phosphate dehydrogenase B (gi|357114230, L93) was also upregulated and peaked at 24 h. In addition, the key Calvin cycle enzyme isocitrate dehydrogenase [NADP] was identified in both roots and leaves (gi|357135759, L143, and R72).

### Detoxification and stress defense

ROS are the toxic and highly reactive by-products of aerobic metabolism^49^. The balance between production and scavenging of ROS can be disrupted by various abiotic and biotic stresses, leading to a rapid and transient increase in intracellular ROS levels^28^. Therefore, plant cells must maintain redox homeostasis and be able activate ROS-scavenging mechanisms to resist oxidative stress. L-ascorbate peroxidase 1 (gi|357112766, L45, and R27) and L-ascorbate peroxidase 2 (gi|357121373, L42, and R25) are key enzymes capable of removing ROS^50^, which were identified from both roots and leaves in this study (Tables S2 and S3). Both proteins were upregulated, peaking after 24–48 h of drought stress and returning to control levels in the roots and leaves of recovering plants. These changes also occur in soybean undergoing drought stress and during post-drought recovery^51^. This suggests that L-ascorbate peroxidase plays an important role in cell survival and drought tolerance. The 2-Cys peroxiredoxin BAS1 (gi|357149358, L33, and R13) was also identified from both roots and leaves in this study. 2-Cys peroxiredoxins are a highly conserved family of enzymes that catalyze the transfer of electrons from sulfhydryl residues to break down peroxides and protect cells from oxidative stress^52^,^53^. Our results demonstrated that the 2-Cys peroxiredoxin BAS1 was rapidly upregulated in response to drought treatment, peaking at 24 h in roots but remaining steady in leaves (Table 1). This suggests that sulfhydryl residue metabolism in roots was more active than in leaves perhaps enhancing plant drought stress resistance. Glutathione S-transferase (R14, R15, and R113) is a key component of the ascorbate recycling system and plays a role in redox homeostasis, particularly in scavenging ROS^54^,^55^. These three DAPs were all upregulated in roots in response to drought stress but were able to return to control levels in recovering seedlings, highlighting their importance in drought resistance. In addition, significant changes of MDA and proline content also reflect the different response of roots and leaves to drought or drought recovery (Fig. 1).

### Protein metabolism

Drought stress can lead to protein misfolding and inactivation, and also to plant cells becoming desiccated. The chaperone protein ClpC1 is a molecular chaperone of the Hsp100 family^56^ which prevents the aggregation of misfolded proteins^57^. This protein (gi|357149201, R143) was upregulated in roots responding to drought stress and peaked at 6 h (Table 1). Therefore, it could play a role in protecting protein structure early in the stress response. Protein R11 is the 40S ribosomal protein S7 (gi|357112663) which is important in protein synthesis. It was upregulated in response to drought treatment but returned to normal levels during the subsequent recovery of seedlings, indicating that protein synthesis in roots was stimulated by drought stress. Protein disulfide-isomerase-like isoform 2 (gi|357157255, R91) is a molecular chaperone containing thioredoxin (TRX) domains that helps to form disulfide bonds during protein folding^58^. It was maintained at high levels in roots in response to drought stress between 24 and 48 h but returned to normal levels in recovering seedlings (Table 1), suggesting it plays a role in protecting protein structure during the later stages of the stress response. The large number of proteins involved in protein synthesis and degradation under drought stress and the subsequent recovery of seedlings suggests that enhanced protein metabolism is essential for plants adapting to drought conditions^59^.

In leaves, the elongation factor Tu (gi|357149925, L99) promotes the GTP-dependent binding of aminoacyl-tRNA to the A-site of ribosomes during protein biosynthesis^60^. Our results demonstrated that elongation factor Tu was downregulated in response to drought stress, suggesting that protein synthesis is inhibited under drought conditions. Chaperonin CPN60-2 (gi|357146493, L118) is implicated in importing mitochondrial proteins and macromolecular assembly. It facilitates the correct folding of imported proteins, prevents misfolding, and promotes refolding and assembly of polypeptides damaged by stress in the mitochondrial matrix. Chaperonin CPN60-2 was downregulated significantly by 24 h of drought treatment compared with the control sample (Table 1), indicating that mitochondrial protein metabolism was inhibited and energy metabolism in leaf tissue was inactive under drought treatment.

### Cell wall and cell structure

Excessive or prolonged stress leads to cellular damage, structural disintegration, and ultimately plant cell death^26^. This study identified six root proteins with structural roles in the plant cell (Table S2). These included actin-3 (gi|357160768, R73) and actin-9 (gi|357135037, R70), which are essential components of the cell cytoskeleton and have important roles in cytoplasmic streaming, cell shape determination, cell division, organelle movement, and extension growth^61^. Drought induced significant upregulation of these proteins and they peaked at 48 h (Table 1), perhaps helping to maintain cell metabolism under these conditions. The tubulin beta-1 (gi|357117233, R83) and beta-3 (gi|357136741, R85) chains are major constituents of microtubules and their accumulation also peaked 48 h after the onset of drought stress. We recently demonstrated that actin-3 and the tubulin beta-3 chain were upregulated in *B. distachyon* roots in response to H_2_O_2_ stress^26^. Root cells are more susceptible to dehydration than leaf cells, so it is important that root cells can strengthen their structure in response to drought stress by generating cytoskeletal proteins. The leaf cell structure may be strengthened by the significant upregulation of two alpha-galactosidases (gi|357146802, L85, and L95) 12 and 24 h after the onset of drought stress.

### A putative synergistic response network regulating responses to drought stress and subsequent recovery in *Brachypodium* roots and leaves

**Figure 8:**
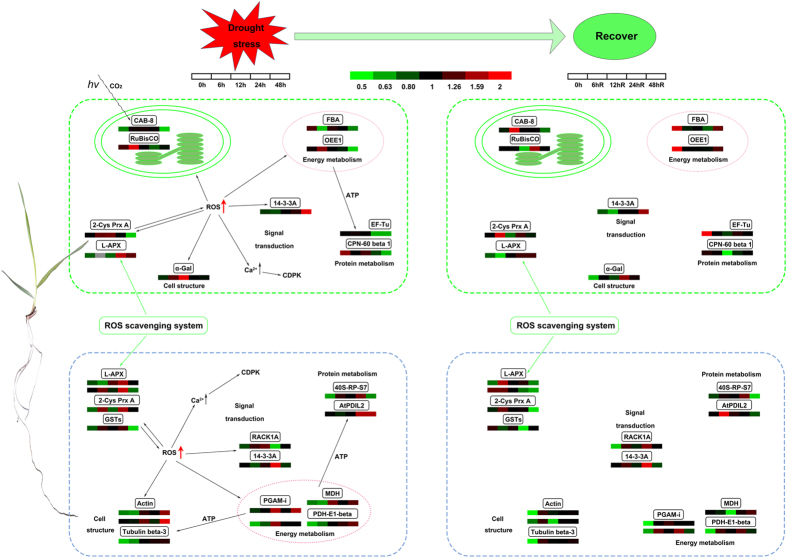
A pathway model of drought stress and recovery responses in Bd21 roots and leaves. 2-Cys Prx A,2-Cys peroxiredoxin BAS1; 40S-RP-S7,40S ribosomal protein S7-like; α-Gal,Alpha-galactosidase-like;Actin,actin-97-like; AtPDIL2,protein disulfide-isomerase-like isoform 2; CAB-8,chlorophyll a-b binding protein 8; CPN-60 beta 1,chaperonin 60 subunit beta 1; EF-Tu,elongation factor Tu; FBA,fructose-bisphosphate aldolase; GSTs,glutathioneS-transferase; L-APX,L-ascorbate peroxidase; MDH,malate dehydrogenase; OEE1,Oxygen-evolving enhancer protein 1; PDH-E1-beta,pyruvate dehydrogenase E1 component subunit beta; PGAM-I,2,3-bisphosphoglycerate-independent phosphoglycerate mutase-like; RuBisCO,ribulose-1,5-bisphosphate carboxylase/oxygenase large subunit; Tubulin beta-3,tubulin beta-3 chain-like.

Using our results and previous studies, we propose a putative synergistic response network for *Brachapodium* roots and leaves responding to drought stress. As shown in Fig. 8, Bd21 seedlings reacting to drought stress rapidly accumulate ROS leading to a series of metabolic changes. First, the root cells produce drought stress signals to generate differential accumulation of signal transduction components. Drought stress causes the root cell ROS concentration to increase, and high concentrations of ROS result in Ca^2+^ stored in organelles being released into the cytoplasm^62^. When the concentration of Ca^2+^ reaches a particular threshold, CDPK is activated^63^. Specific 14-3-3 proteins can also activate CDPK^43^, which in turn activates transcription factors that induce or repress gene expression. As a result, plants are able to produce appropriate responses to the stress conditions^44^. During prolonged drought stress, the ROS scavenging system is activated and proteins regulating redox balance that include GSTs and L-APX are upregulated. These proteins can break down excessive intracellular ROS and restore the normal intracellular redox environment. Drought stress can lead to protein misfolding, which activates protein metabolism in roots. This leads to a significant increase in the accumulation of factors involved in protein synthesis, including 40S-RP-S7 and AtPDIL2. If the drought period is prolonged, proteins involved in cell structure maintenance accumulate. Proteins with important functions in energy metabolism are continuously upregulated to generate ATP and allow plants to perform vital metabolic functions and to combat stress. During the recovery period, the abundance of most proteins returns to normal, but high levels of some are maintained. These include proteins involved in cell structure maintenance, such as actin and tubulin beta-3.

When the stress signal reaches leaves, ROS also accumulate leading to increased intracellular Ca^2+^ concentrations, CDPK activation, and triggering signaling cascades. Subsequently, the ROS scavenging system is activated to restore normal leaf cell redox conditions. Factors involved in protein metabolism that include EF-Tu and CPN-60 beta 1 are downregulated and leaf protein metabolism is inhibited. At the same time, proteins involved in cell structure maintenance are upregulated in response to drought. In addition, because of the effect of drought stress on energy metabolism, proteins involved in photosynthesis and energy metabolism are downregulated. During recovery from drought, most proteins return to their normal levels, except for those related to cell structure and stress responses. During the advanced stages of drought stress, leaves curled and root tips gradually became darker in color. The clearest difference between roots and leaves responding to drought stress is the inhibition of photosynthesis that occurs specifically in leaves.

## Conclusion

An integrated proteomic analysis of Bd21 roots and leaves combined with plant physiology, biochemistry, and gene expression studies revealed that synergistic response is active during drought stress and subsequent plant recovery. When subjected to drought, leaves were more sensitive than roots and seedling morphology changed significantly. Some physiological changes were irreversible if the drought period exceeded 24 h. Energy and protein metabolism are stimulated in roots responding to drought stress but inhibited in leaves. Drought significantly inhibits photosynthesis in leaves. In both roots and leaves, 14-3-3-like protein A played a key role in the synergistic response to drought stress defined by our PPI network analysis. The key to understanding the signal transduction processes involved in the response to drought stress may be found in the crosstalk pathways that connect roots and leaves under these conditions. Our results provide new insight into the molecular mechanisms plants adaptation to tolerate drought stress.

## Materials and Methods

### Plant material, seedling cultivation and drought treatments

Seeds of *B. distachyon* Bd21, kindly provided by Dr. John Vogel from USDA-ARS, were sterilized and cultured as mentioned in previous study^33^. The treatment process of seedling is shown in Fig. S5, and both control and treated groups included three biological replicates. At the three leaf stage, PEG effects was imposed by adding 20% (w/v) polyethylene glycol (PEG) 6000 to the Hoagland solution to obtain an osmotic potential (ψs) of –0.75 MPa in plastic containers. The water potential was measured by a vapor pressure osmometer (Wescor Vapro 5520, USA) according to previous methods^25^. The seedlings of treatment groups were treated with PEG effects for 6, 12, 24 and 48 h, respectively, half of the seedling roots and leaves were harvested for further analyses. After the stress treatment, the seedlings of treatment groups in three biological replicates were transferred to Hoagland solution without PEG to recover for 48 h, and then roots and leaves were collected. The samples before treated with PEG were used as a control. All the samples were snap-frozen in liquid nitrogen and then stored at −80 °C for later use. A flow chart of the complete experimental design is presented in Fig. S6.

### Phenotype and physiological parameter measurement

Seedling phenotype under control, drought treated and recovery groups was observed, and plant height and main root length were measured. The fresh roots and leaves were used to measure the physiological parameters. Chlorophyll content, relative water content (RWC), malondialdehyde (MDA) and proline content were measured according to Lv *et al*.^33^ and Hao *et al*.^12^. Three biological replicates were used to minimize experimental error.

### Protein extraction and 2D-DIGE

Root and leaf total protein from three biological replicates was extracted according to Cascardo *et al*.^64^ with minor modifications. Through preliminary experiment we found that 24 h treatment is a node for Bd21 under 20% PEG drought condition. Thus, we choose control, 24 h and 24 hR to do two-dimensional difference gel electrophoresis (2D-DIGE) analysis for differential accumulation protein (DAP) identification based on Rollins *et al*.^65^ and the experimental design was listed in Table S1.

Protein extracts were minimally labeled with fluorescent Cy2, Cy3, or Cy5 N-hydroxysuccinimide (NHS) esters (Lumiprobe, LLC). Protein solutions were diluted to 5 μg/μl with labeling buffer (30 mM Tris, 7 M urea, 2 M thiourea, 4% CHAPS, pH of 8.5) and 1 μl of working solution containing 400 picomoles of fluorescent ester was added to 50 μg of protein. The protein and dye solution was vortexed and centrifuged before keeping on ice in the dark for 30 min. To quench any remaining unreacted esters 1 μl of 10 mM lysine was added to the reaction and the tube was vortexed and centrifuged before keeping on ice in the dark for 10 min. The labeled protein solution was then subsequently used for 2D-DIGE. Cy2 was exclusively used for labeling of pooled internal standards consisting of an equal mixture of all protein samples used in a given experiment; dyes Cy3 and Cy5 were used on individual samples as shown in Table S1. Two dimensional gels containing protein labeled with Cy2, Cy3 and Cy5 NHS esters were imaged at a 100 μm resolution with the Typhoon TM 9400 (GE Healthcare Life Science) using long pass filters for either 520 nm 580 nm or 670 nm. Photomultiplier tube gain voltage was adjusted for each Cy dye for each gel to get clear 2D-DIGE images. DeCyder 2D Software V6.5 was used to analyze the images and only those with significant and biological reproducible changes (abundance variation at least 2-fold, Student’s t-Test, *p* < 0.05) were considered to be DAP spots.

### 2-DE

After 2D-DIGE, 2-DE was used to test the dynamic changes of the identified DAP spots under different drought treated times based on Lv *et al*.^33^. Three biological replicates were conducted for 2-DE. Each sample including 1 mg total protein in 360 μL rehydration buffer(7 M urea, 2 M thiourea, 2% w/v CHAPS, 0.2% bromophenol blue) containing 65mMDTT and 0.5% IPG buffer (pH 4–7) (GE Healthcare) was loaded onto a 18 cm, pH 4–7 linear gradient IPG strip (GE Healthcare). After electrophoresis, all gels were stained with Coomassie brilliant blue (CBB). Image analysis was performed with ImageMaster 2D Platinum Software Version 7.0 (Amersham Biosciences). The abundance of each spot was estimated by the percentage volume (%Vol). Only those with significant and biological reproducible changes (abundance variation at least 2-fold, Student’s t-Test, p b 0.05) were considered to be DAP spots.

### Statistical analysis

The coordinately accumulated DAP spots were to perform hierarchical clustering analysis by Cluster 3.0 software according to the method described by Eisen *et al*.^66^. The relative ratios of DAP spots were conducted log2 transforming, and then the Euclidean distance similarity metric was used to define the similarity and the hierarchical clusters were assembled using the complete-linkage clustering method. Cluster 3.0 allows for clustering result visualization with a dendrogram of the DAP spots.

Principal component analysis (PCA) is a method that finds the main variations and reveals hidden structures present in the data set. In this study, coefficient and KMO and Bartlett’s test of sphericity were selected for dimension reduction analysis and the results were displayed in the loading plot and scatter plot, respectively. The loading plot and scatter plot of PCA was calculated or displayed with the average center value of each DAP spot. PCA was performed using SPSS 19.0 software.

### Protein identification using MALDI-TOF/TOF-MS

The selected protein spots were manually excised from the 2-DE gels and digested with trypsin in centrifuge tubes (1.5 mL). Briefly, gel slices were destained two to three times with 100 μL bleaching solution (50% 25 mM NH_4_HCO_3_ and 50% ACN) until the slices were colorless. The samples were agglomerated and mixed with 100 μL ACN. The digestion was performed at 37 °C for 16 h with 20 μL diluted solvent (50 m MNH_4_HCO_3_) containing 50 ng trypsin enzyme (Promega, USA). The digested peptides were extracted three times with 0.1% TFA in 60% ACN at 37 °C for 1 h and the supernatant containing peptides was dehydrated in a vacuum. The freeze-dried peptides were dissolved in 50% ACN and 0.1% TFA containing 5 mg/mL CHCA. Identification of the spots was performed by using matrix-assisted laser desorption/ionization time-of-flight/time-of-flight mass spectrometry (MALDI-TOF/TOF-MS) as described by Lv *et al*.^33^. The MS together with MS/MS spectra were searched against the NCBI Brachypodium protein database (25,559 entries in total, downloaded on March 7, 2014) using software MASCOT version 2.1 (Matrix Science) with the following parameter settings: trypsin cleavage, one missed cleavage allowed, carbamidomethylation set as fixed modification, oxidation of methionines allowed as variable modification, peptide mass tolerance set to 100 ppm and fragment tolerance set to ±0.3 Da. All searches were evaluated based on the significant scores obtained from MASCOT. The Protein Score C. I.% and Total Ion Score C. I.% were both set above 95% and the significance threshold p < 0.05 for the MS/MS.

### Bioinformatics analysis

Protein function classification was based on the annotation from UniProt^67^. The subcellular localization was the integration of prediction results through WoLF PSORT (http://www.genscript.com/wolf-psort.html), Plant-mPLoc (http://www.csbio.sjtu.edu.cn/bioinf/plant-multi/)^68^ and UniprotKB. The Search Tool for the Retrieval of Interacting Genes/Proteins (STRING) database of physical and functional interactions^69^ was used to analyze the PPI. The sequences of all identified proteins were blasted in the National Center for Biotechnology Information (NCBI) clusters of euKaryotic Orthologous Groups (KOG) database (http://eggnog.embl.de/version_3.0/) to obtain the protein KOG numbers^70^ (Tables S2 and S3), which was used for the production of PPI network and then displayed by Cytoscape (version 3.0.0) software^71^,^72^.

### RNA extraction and qRT-PCR

qRT-PCR was used to measure the transcript levels of the protein of interest in roots and leaves. Total RNA was isolated from the roots and leaves of Bd21 using TRIZOL Reagent (Invitrogen). Then Reverse transcription reactions were performed with the PrimeScript^®^ RT Reagent Kit with gDNA Eraser (TaKaRa, Shiga, Japan) according to the manufacturer’s instructions. Gene-specific primers were designed using online Primer3Plus (Table S4) and their specificities was checked by observing the melting curve of the RT-PCR products and the specific band on the agarose gel. qRT-PCR was performed in a 20 μL volume containing 10 μL 2×SYBR^®^ Premix Ex Taq™ (TaKaRa, Shiga, Japan), 2 μL 50-fold diluted cDNA, 0.15 μL of each gene-specific primer, and 7.2 μL ddH2O. PCR conditions were the following: 95 °C for 3 min, 39 cycles of 15 s at 94 °C, 61 °C for 15 s, and 72 °C for 10 s, a melt curve of 65 to 95 °C. The two standard curve relative quantitation method was used to analyze gene transcript expression profiles and three biological replicates were used for each sample^73^. Reactions were conducted on a CFX96 Real-time PCR Detection System (Bio-Rad). All data were analyzed with CFX Manager Software (Bio-Rad)^33^.

## Additional Information

**How to cite this article:** Bian, Y. *et al*. Integrated proteomic analysis of *Brachypodium distachyon* roots and leaves reveals a synergistic network in the response to drought stress and recovery. *Sci. Rep.* **7**, 46183; doi: 10.1038/srep46183 (2017).

**Publisher's note:** Springer Nature remains neutral with regard to jurisdictional claims in published maps and institutional affiliations.

## Supplementary Material

Supplementary Figures

Supplementary Tables

## Figures and Tables

**Table 1:** Representative differentially accumulated proteins identified by MALDI-TOF/TOF-MS.

Spot No.	Protein Name	Accession No.	Protein function classification	Average% Vol. Ratio 0 h:6 h:12 h:24 h:48 h:6 hR:12 hR:24 hR:48 hR	KOG No	P-value	Subcellular Localizatin
L54	14-3-3-like protein A-like	gi|357118054	Signal transduction	1:0.97:1.11:1.19:1.6:0.81:1.23:1.23:1.75	KOG0841	0.017	Plastid
R136	14-3-3-like protein A-like	gi|357118054	Signal transduction	1:0.71:1.2:2.6:0.79:1.26:0.9:1.96:0.8	KOG0841	0.021	Plastid
R45	guanine nucleotide-binding protein subunit beta-like protein A-like	gi|357132654	Signal transduction	1:1.77:2.13:0.58:1.37:1.63:1.36:1.85:1.45	KOG0279	0.013	Cyto
R92	2,3-bisphosphoglycerate-independent phosphoglycerate mutase-like	gi|357125604	Energy metabolism	1:1.7:2.68:1.93:2.03:2.21:1.51:1.61:2.76	KOG4513	0.015	Cyto
R95	2,3-bisphosphoglycerate-independent phosphoglycerate mutase-like	gi|357125604	Energy metabolism	1:1.21:2.25:1.21:2.25:1.46:1.6:1.45:1.47	KOG4513	0.034	Cyto
R96	2,3-bisphosphoglycerate-independent phosphoglycerate mutase-like	gi|357125604	Energy metabolism	1:1.24:2.1:1.52:1.54:1.57:1.32:1.67:1.17	KOG4513	0.022	Cyto
R57	malate dehydrogenase, cytoplasmic-like	gi|357146638	Energy metabolism	1:0.92:1.79:1.35:1.66:0.91:0.54:1.07:1.23	KOG1496	0.022	Cyto
R60	pyruvate dehydrogenase E1 component subunit beta, mitochondrial-like	gi|357148637	Energy metabolism	1:1.45:2.74:3.55:4.81:2.37:1.94:2.53:1.7	KOG0524	0.039	Mito
L91	Phosphoglycerate kinase, chloroplastic-like isoform 1	gi|357133147	Energy metabolism	1:1.31:0.99:0.79:0.63:0.55:0.52:0.82:0.76	KOG1367	0.019	Plastid
L93	glyceraldehyde-3-phosphate dehydrogenase B, chloroplastic-like	gi|357114230	Energy metabolism	1:0.72:1.18:2.03:1.49:1.7:1.15:1.09:2.08	KOG0657	0.044	Plastid
L143	isocitrate dehydrogenase [NADP], chloroplastic-like	gi|357135759	Energy metabolism	1:0:1.88:1.8:0.61:2.11:1.27:2.87:1.27	KOG1526	0.018	Cyto
R72	isocitrate dehydrogenase [NADP], chloroplastic-like	gi|357135759	Energy metabolism	1:0.41:1.39:0.71:1.33:1.09:0.53:0.93:0.93	KOG1526	0.033	Cyto
L138	Chlorophyll a-b binding protein 2, chloroplastic-like isoform 6	gi|357159913	Energy metabolism	1:2.9:2.45:5.15:3.46:1.43:2.11:2.01:2.28		0.041	Plastid
L35	chlorophyll a-b binding protein 8,chloroplastic-like	gi|357139429	Energy metabolism	1:1.61:1.65:1.53:0.82:1.89:1.1:1.09:0.8		0.026	Plastid
L15	ribulose-1,5-bisphosphate carboxylase/oxygenase large subunit	gi|194033157	Energy metabolism	1:1.37:0.86:0.71:0.83:0.98:0.64:1.42:0.99		0.018	Plastid
L144	ribulose-1,5-bisphosphate carboxylase/oxygenase large subunit	gi|194033157	Energy metabolism	1:0:1.58:0.7:0.47:0:0:1.06:0		0.033	Plastid
L113	ribulose-1,5-bisphosphate carboxylase/oxygenase large subunit	gi|194033157	Energy metabolism	1:1.15:1.22:1.38:0.24:0.46:0:1.13:0.72		0.035	Plastid
L45	L-ascorbate peroxidase 1, cytosolic-like	gi|357112766	Detoxification and stress defense	1:0:0.98:2.65:1.96:0.79:1.21:1.37:1.38		0.042	Cyto
R27	L-ascorbate peroxidase 1, cytosolic-like	gi|357112766	Detoxification and stress defense	1:1.4:0.89:1.79:0.72:1:0.85:0.7:0.64		0.015	Cyto
L42	L-ascorbate peroxidase 2, cytosolic-like	gi|357121373	Detoxification and stress defense	1:0.51:0.73:0.9:1.46:1.47:0.72:1.29:1.75		0.006	Cyto
R25	L-ascorbate peroxidase 2, cytosolic-like	gi|357121373	Detoxification and stress defense	1:0.85:1.47:1.79:1.2:1.42:1.15:1.21:0.98		0.029	Cyto
L33	2-Cys peroxiredoxin BAS1,chloroplastic-like	gi|357149358	Detoxification and stress defense	1:1.08:1.09:1:0.86:1.76:0.74:1.2:0.92	KOG0852	0.043	Plastid
R13	2-Cys peroxiredoxin BAS1,chloroplastic-like	gi|357149358	Detoxification and stress defense	1:1.56:1.02:1.67:1.22:1.32:1.08:1.12:0.69	KOG0852	0.022	Plastid
R14	glutathione S-transferase 3-like	gi|357126684	Detoxification and stress defense	1:1.45:1:0.93:0.38:0.66:0.75:0.62:0.63	KOG0867	0.031	Cyto
R15	glutathione S-transferase DHAR2-like isoform 1	gi|357134821	Detoxification and stress defense	1:1.5:1.28:1.56:0.73:0.82:0.96:0.64:0.92	KOG1422	0.027	Cyto
R113	glutathione S-transferase DHAR2-like isoform 1	gi|357134821	Detoxification and stress defense	1:1.34:1.67:1.36:0.79:0.66:1.03:0.55:0	KOG1422	0.016	Cyto
R143	chaperone protein ClpC1,chloroplastic-like	gi|357149201	Protein metabolism	1:1.82:1.41:1.42:0:1.11:1.17:1.23:0	KOG1051	0.018	Plastid
R11	40S ribosomal protein S7-like	gi|357112663	Protein metabolism	1:1.78:1.53:2.09:1.06:1.38:1.26:1.8:0.64	KOG3320	0.044	Cyto
R91	protein disulfide-isomerase-like isoform 2	gi|357157255	Protein metabolism	1:0.92:1.01:1.49:1.47:1.7:1.11:1:0.82	KOG0190	0.039	ER
L99	elongation factor Tu, chloroplastic-like	gi|357149925	Protein metabolism	1:1.09:1:0.47:0.47:0.73:0.67:0.77:0.73	KOG0460	0.039	Plastid
L118	Chaperonin CPN60-2, mitochondrial-like	gi|357146493	Protein metabolism	1:0.83:0.75:0.39:0.53:0.58:0.33:0.81:0.64	KOG0356	0.037	Mito
R73	actin-3-like	gi|357160768	Cell wall and cell structure	1:0.92:1.52:0.85:2.43:1.79:0.75:1.51:1.49	KOG0676	0.039	Cyto
R70	actin-97-like	gi|357135037	Cell wall and cell structure	1:0.91:2.22:1.76:3.23:2.32:1.83:1.98:2.03	KOG0676	0.013	Cyto
R83	tubulin alpha-1 chain-like	gi|357117233	Cell wall and cell structure	1:0.88:0.8:0.71:1.41:1.37:0.54:1:0.6	KOG1376	0.035	Cyto
R85	tubulin beta-3 chain-like	gi|357136741	Cell wall and cell structure	1:1.3:2.57:2.96:3.19:3.49:2.61:2.71:3.08	KOG1375	0.04	Cysk
L85	Alpha-galactosidase-like	gi|357146802	Cell wall and cell structure	1:1.81:3.29:1.24:1.15:1.55:1.32:2.15:1.65	KOG2366	0.042	ER
L95	Alpha-galactosidase-like	gi|357146802	Cell wall and cell structure	1:0:0.92:2.5:2.01:1.55:1.9:1.94:2.24	KOG2366	0.03	ER
