# Supplementary material for: Integrated proteomic analysis of Brachypodium distachyon roots and leaves reveals a synergistic network in the response to drought stress and recovery
Source: Sci Rep. 2017 Apr 7;7:46183. doi: 10.1038/srep46183 (PMC5384013; doi:10.1038/srep46183)
Supplement: Supplementary Figures [file srep46183-s1.pdf]

Integrated proteomic analysis of *Brachypodium distachyon* roots and leaves reveals a synergistic network in the response to drought stress and recovery

Yanwei Bian[a], Xiong Deng[a], Xing Yan[a], Jiaxing Zhou,

Linlin Yuan, and Yueming Yan[*]

Figure S1. Morphological changes in Bd21 roots and leaves under drought stress and recover. CK represents the control; 6 h, 12 h, 24 h and 48 h represent seedlings at 6, 12, 24, and 48 h treatment under drought, respectively; 6 hR, 12hR, 24 hR and 48 hR represent seedlings at 6, 12, 24, and 48 h treatment under drought and experience recovery process for 48h, respectively.

Figure S2. Two-dimensional electrophoresis maps of Bd21 root proteome under drought stress and recovery. A total of 78 DAP spots screened out from 2-DE maps of leaf are highlighted.

Figure S3. Two-dimensional electrophoresis maps of Bd21 leaf proteome under drought stress and recovery. A total of 98 DAP spots screened out from 2-DE maps of leaf are highlighted.

Figure S4. Protein expression clustering analysis of DAP spots from 2-DE maps of root and leaf. (A) Hierarchical clustering of DAP spots from roots; (B) Hierarchical clustering of DAP spots from leaves. Each column represents samples from treatment under drought stress and recovery group. Each row displays the change of a DAP spot using color-coding based on the relative ratio. The grey color represents missing value at that time point.

Figure S5. Sketch map of drought treatment and recovery of Bd21.

Figure S6. A flow chart of the complete experimental design.

Figure S1:

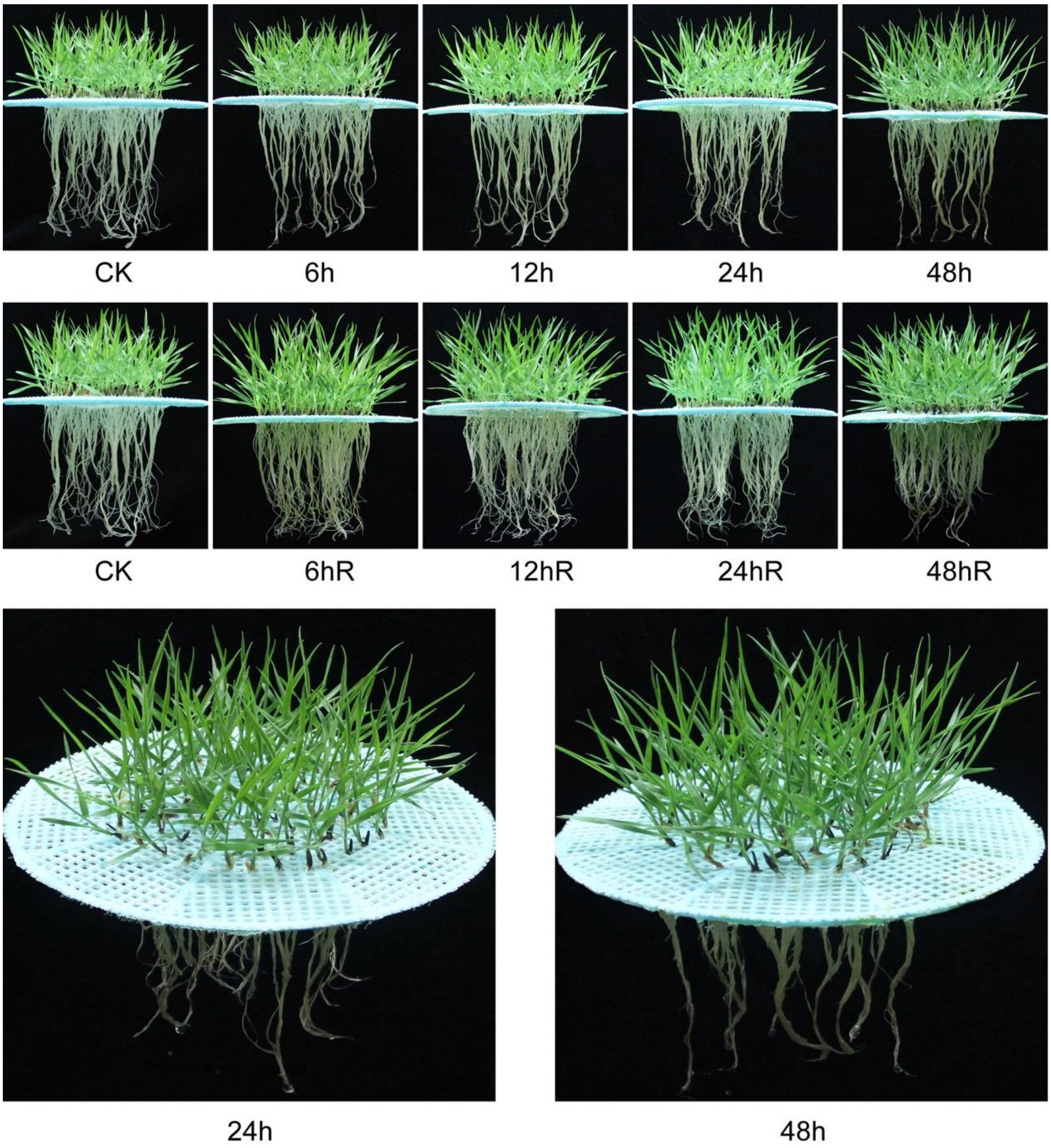

Figure S2:

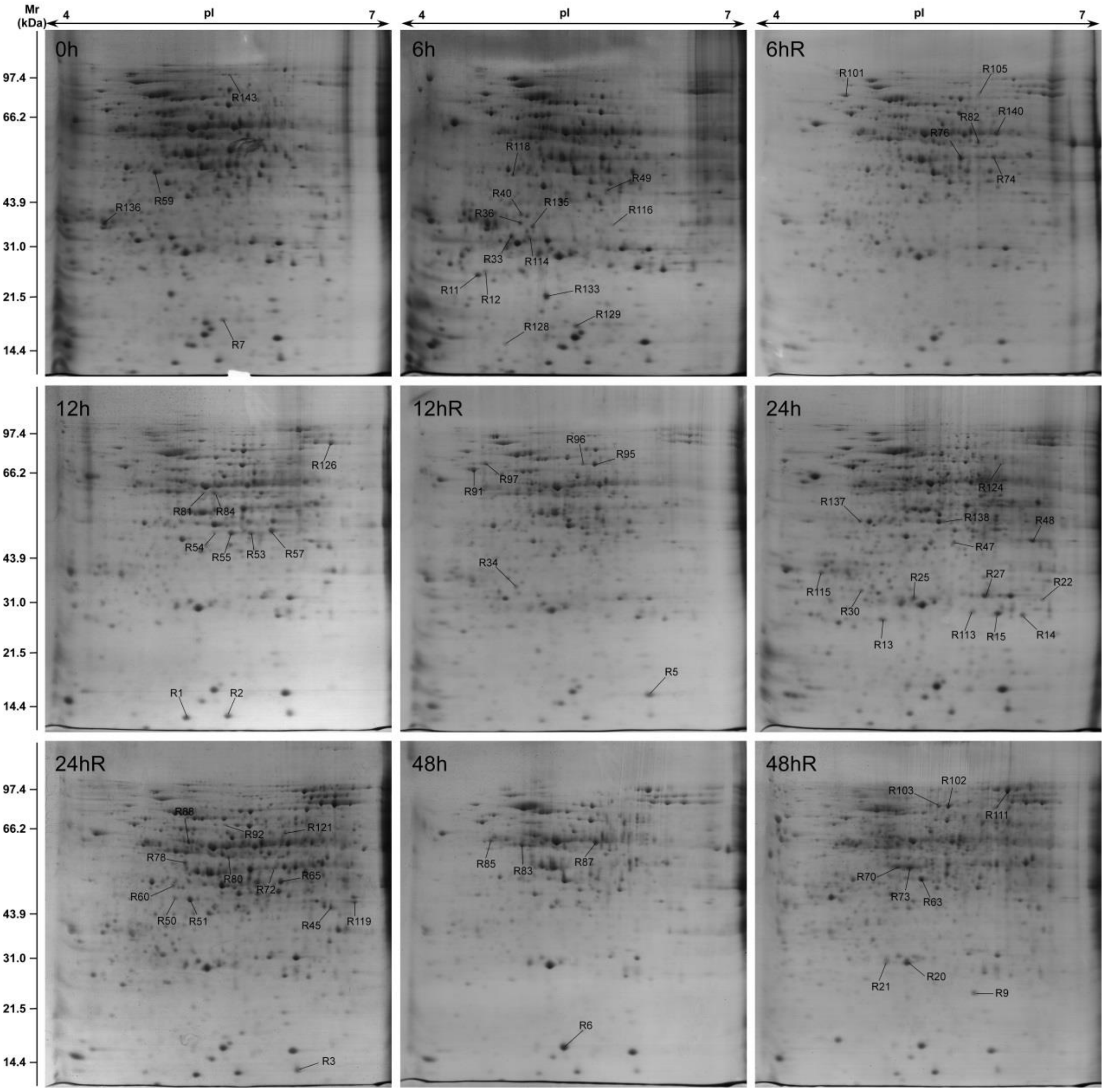

Mr (kDa)
4
pI
7
97.4
66.2
43.9
31.0
21.5
14.4
0h
R143
R59
R136
R7
6h
R118
R49
R40
R135
R36
R116
R33
R114
R11
R12
R133
R129
R128
6hR
R101
R105
R82
R140
R76
R74
12h
R126
R81
R84
R54
R55
R53
R57
R1
R2
12hR
R96
R95
R97
R91
R34
R5
24h
R124
R137
R138
R48
R47
R115
R25
R27
R22
R30
R13
R113
R15
R14
24hR
R88
R92
R121
R78
R80
R65
R72
R60
R50
R51
R45
R119
R3
48h
R85
R83
R87
R6
48hR
R103
R102
R111
R70
R73
R63
R21
R20
R9

Figure S3:

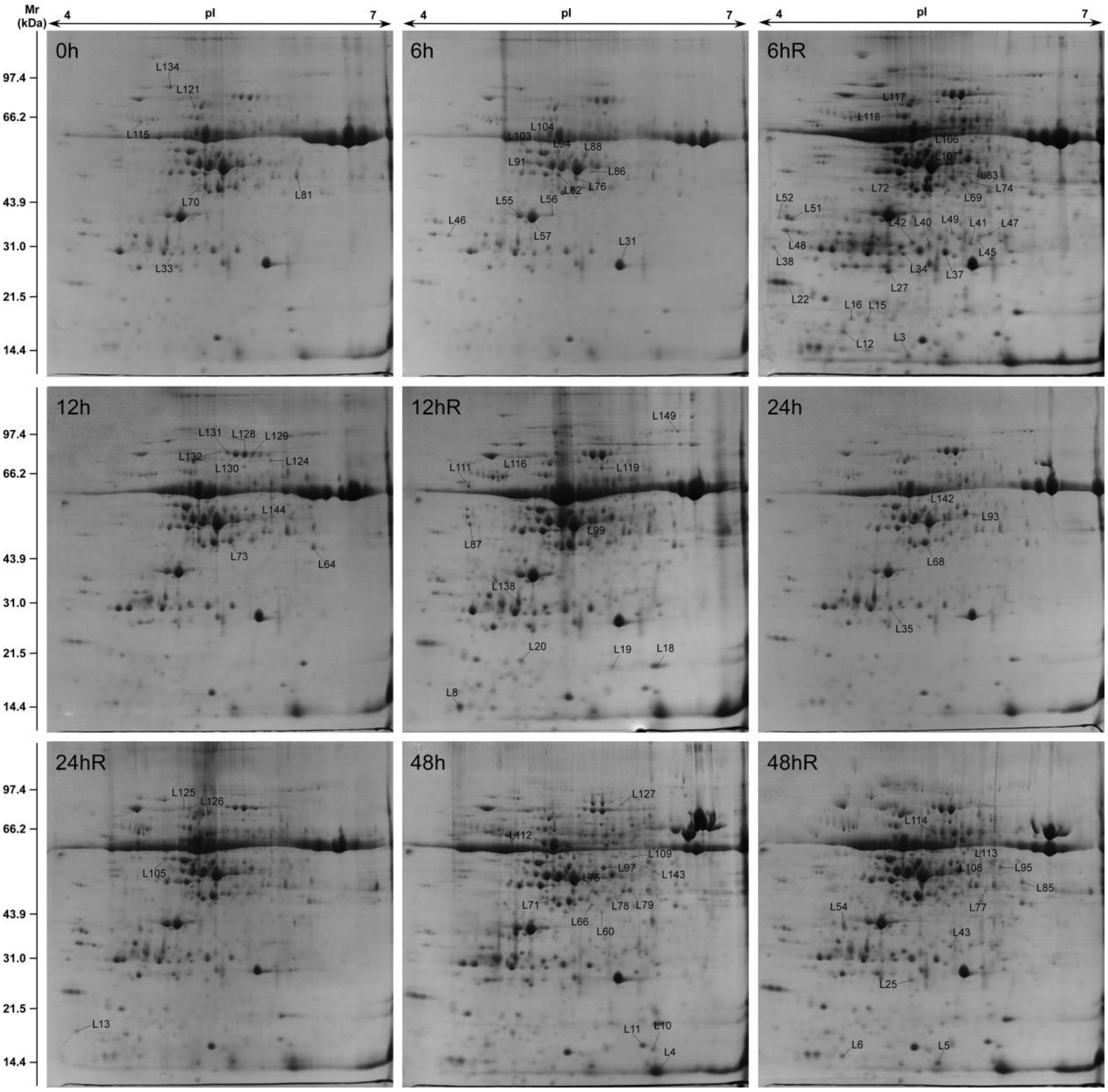
Mr
(kDa)
4
pI
7
97.4
66.2
43.9
31.0
21.5
14.4
0h
L134
L121
L115
L81
L70
L33
6h
L104
L103
L94
L88
L91
L86
L82
L76
L55
L56
L46
L57
L31
6hR
L117
L118
L106
L107
L83
L72
L69
L74
L52
L51
L42
L40
L49
L41
L47
L48
L45
L38
L34
L37
L27
L22
L16
L15
L12
L3
12h
L131
L128
L129
L132
L124
L130
L144
L73
L64
12hR
L149
L111
L116
L119
L89
L87
L138
L20
L19
L18
L8
24h
L142
L93
L68
L35
24hR
L125
L126
L105
L13
48h
L127
L112
L109
L97
L143
L75
L71
L78
L79
L66
L60
L11
L10
L4
48hR
L114
L113
L108
L95
L85
L54
L77
L43
L25
L6
L5

Figure S4:

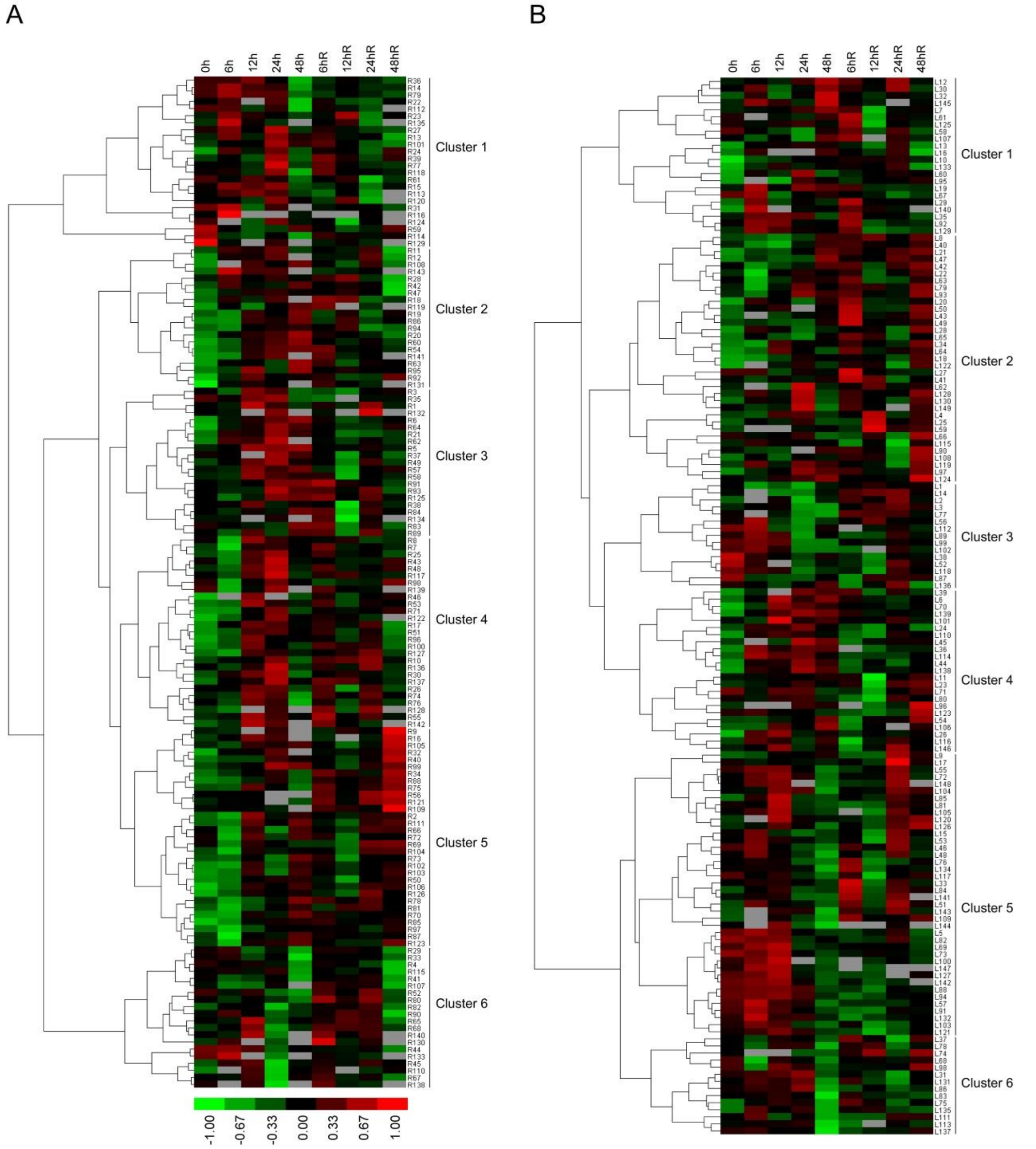
A
B
0h
6h
12h
24h
48h
6hR
12hR
24hR
48hR
Cluster 1
Cluster 2
Cluster 3
Cluster 4
Cluster 5
Cluster 6
-1.00
-0.67
-0.33
0.00
0.33
0.67
1.00

Figure S5:

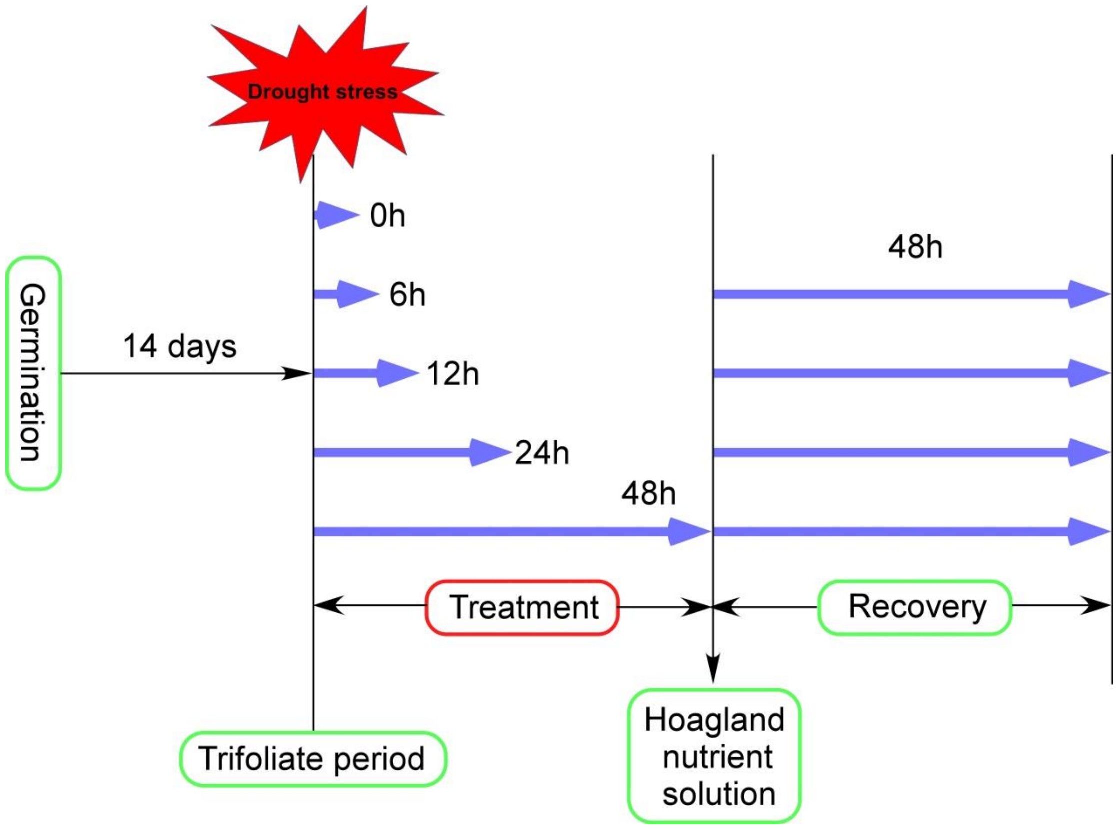
Drought stress
0h
6h
12h
24h
48h
48h
Germination
14 days
Treatment
Recovery
Trifoliate period
Hoagland nutrient solution

Figure S6:

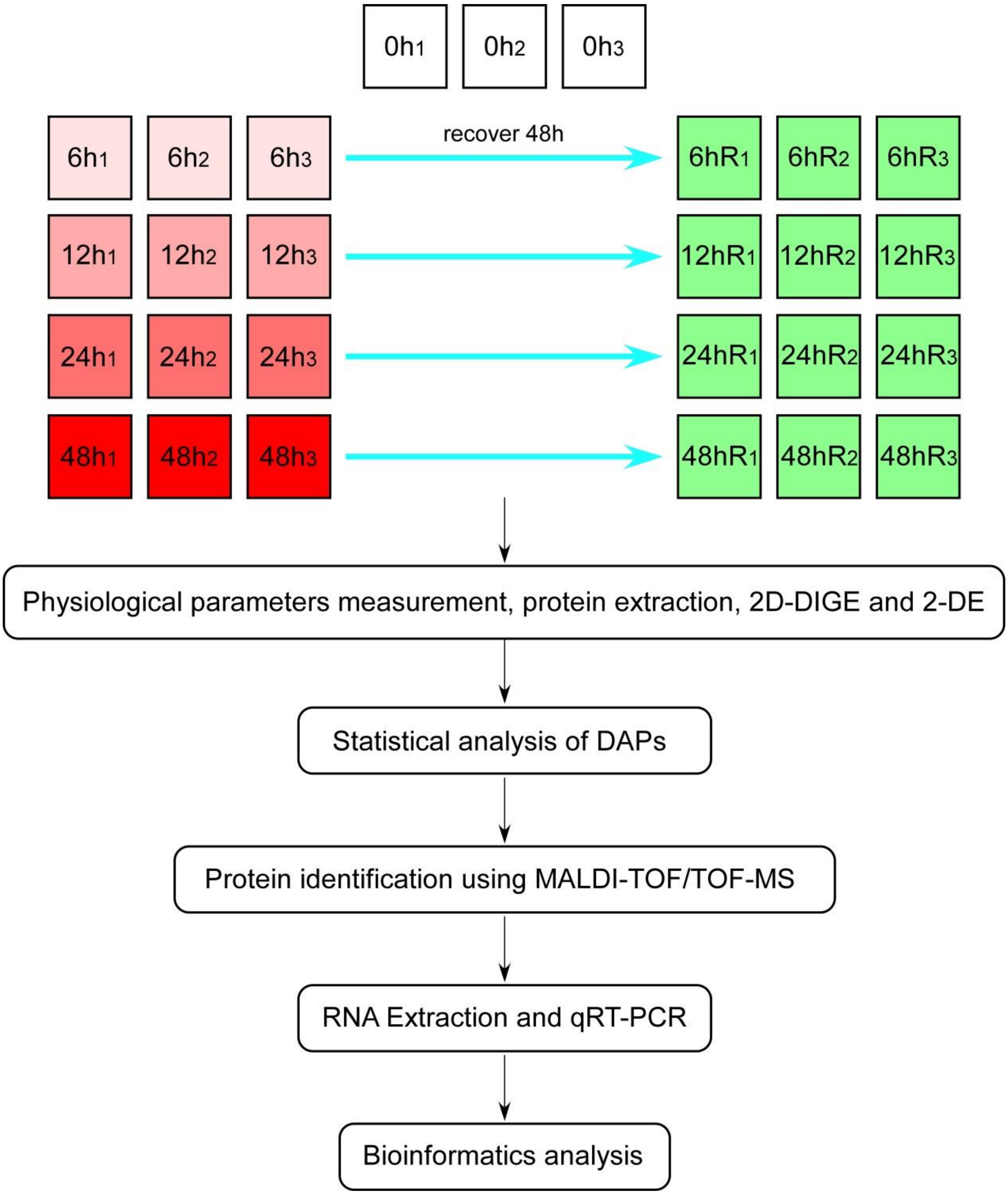
0h1
0h2
0h3
6h1
6h2
6h3
recover 48h
6hR1
6hR2
6hR3
12h1
12h2
12h3
12hR1
12hR2
12hR3
24h1
24h2
24h3
24hR1
24hR2
24hR3
48h1
48h2
48h3
48hR1
48hR2
48hR3
Physiological parameters measurement, protein extraction, 2D-DIGE and 2-DE
Statistical analysis of DAPs
Protein identification using MALDI-TOF/TOF-MS
RNA Extraction and qRT-PCR
Bioinformatics analysis
